# Diagnostic challenges and case management of the first imported case of *Plasmodium knowlesi* in Sri Lanka

**DOI:** 10.1186/s12936-017-1776-1

**Published:** 2017-03-21

**Authors:** A. Dewanee Ranaweera, Manjula N. Danansuriya, Kusumawathie Pahalagedera, W. M. Kumudunayana T. de A. W. Gunasekera, Priyani Dharmawardena, Keng Wai Mak, Pei-Sze Jeslyn Wong, Mei-Zhi Irene Li, Cheong Huat Tan, Hapuarachchige C. Hapuarachchi, Hema D. B. Herath, Deepika Fernando

**Affiliations:** 1 Anti Malaria Campaign, 555/5, Public Health Complex, Narahenpita, Colombo 5, Sri Lanka; 2 Regional Office, Anti Malaria Campaign, Dutugemunu Mawatha, Watapuluwa, Kandy, Sri Lanka; 3 Environmental Health Institute, National Environmental Agency, 11 Biopolis Way, #06-05/08, Singapore, 138667 Singapore; 4 Department of Parasitology, Faculty of Medicine, University of Colombo, Colombo, Sri Lanka

**Keywords:** Malaria, *Plasmodium knowlesi*, Sri Lanka, Imported, Surveillance

## Abstract

**Background:**

Sri Lanka has achieved ‘malaria-free’ status and is now in the phase of prevention of re-introduction of malaria. Imported malaria remains a challenge to resurgence of the disease. The diagnostic challenges encountered and the rapid response initiated to manage a *Plasmodium* infection, which was later confirmed as *Plasmodium knowlesi,* the first reported case from Sri Lanka, is discussed.

**Case presentation:**

An army officer who returned from Malaysia in October 2016 was found to be positive for *Plasmodium* both by microscopy and rapid diagnostic test (RDT) by the Anti Malaria Campaign Sri Lanka (AMC) during his third visit to a health care provider. Microscopy findings were suspicious of *P. knowlesi* infection as the smears showed parasite stages similar to both *Plasmodium malariae* and *Plasmodium falciparum*. Nested PCR at AMC confirmed *Plasmodium* genus, but not the species. In the absence of species confirmation, the patient was treated as a case of *P. falciparum*. The presence of *P. knowlesi* was later confirmed by a semi-nested PCR assay performed at the Environmental Health Institute, National Environmental Agency in Singapore. The parasite strain was also characterized by sequencing the circumsporozoite gene. Extensive case investigation including parasitological and entomological surveillance was carried out.

**Conclusions:**

*Plasmodium knowlesi* should be suspected in patients returning from countries in the South Asian region where the parasite is prevalent and when blood smear results are inconclusive.

## Background

The World Health Organization (WHO) certified Sri Lanka free of malaria in September 2016. As the country moves towards the phase of prevention of re-introduction, imported infections become increasingly significant as high densities of the predominant vector, *Anopheles culicifacies* are being reported in most parts of the country and the population remains at-risk of developing malaria [1]. The fact that 221 cases of imported malaria have been reported in Sri Lanka between 2013 and 2016 is important in the context that imported malaria has hampered elimination efforts in Zanzibar [2], which is progressing towards elimination, and in countries which have previously achieved elimination such as Greece and Turkmenistan [3, 4].

Since 2008, when a malaria case is reported in Sri Lanka, information gathered through rigorous case investigation is reviewed thoroughly to exclude any probability of the case being acquired locally in Sri Lanka. Thus far, imported malaria cases that have been reported in the country have been due to *Plasmodium falciparum, Plasmodium vivax, Plasmodium ovale,* and *Plasmodium malariae* all of which have long been recognized to infect humans in nature. This publication discusses the diagnostic challenges and case management of the first imported case of *Plasmodium knowlesi*, the fifth species of human malaria parasite, reported in Sri Lanka. *Plasmodium knowlesi* is primarily a malaria species of non-human primate origin [5–7].

Following the first case of *P. knowlesi* reported in 1965 in an American army personnel who spent five days in a primary forest in the northern part of Peninsular Malaysia [8], a greater interest was paid to this parasite globally after a large focus was highlighted from the Kapit Division of Sarawak, Malaysian Borneo in 2004 [9]. Since then, there have been widespread reports of *knowlesi* malaria from humans [10]. In *P. knowlesi* infections, high parasitaemias can develop rapidly and there is a risk of developing severe disease [11, 12].

## Results

### Patient presentation and investigations

A 31-year old Army Officer who returned on the 31st of October 2016 following five months of military training in Pulada jungles, Johor Bahru, Malaysia, presented to a private medical health care institute in Kandy, situated in the Central Province on the 5th of November 2016 with a 10-day history of fever, chills and rigors, headache and arthralgia. The patient was clinically stable at presentation with no significant physical findings other than a temperature of 103 °F [°C = 39.4]. He had not taken chemoprophylaxis for malaria.

Fever had developed prior to his departure from Malaysia on the 26th of October. Following his return, he had taken treatment from two peripheral government hospitals where he was managed as having viral fever and malaria had not been suspected in the differential diagnosis. It was after his admission to a private hospital in Kandy, 5 days after his arrival, that a consultant physician suspected malaria. The rapid diagnostic test (RDT; HRP2/pLDH Combo test) performed at the private hospital indicated a positive band for the control and pLDH antigen (pan-*Plasmodium* genus specific). Even though the HRP2 band which is specific for *P. falciparum* was not seen in the RDT, the blood smear was reported as *P. falciparum* by a trained medical laboratory technician. The case was notified to the Regional Malaria Officer (RMO) and the Anti Malaria Campaign Sri Lanka (AMC) headquarters. Based on the RDT and microscopy results of regional and central level public health laboratory technicians, and the travel history of the patient, *P. knowlesi* infection was suspected at both laboratories. At regional level early and late trophozoites of *Plasmodium* species (parasitaemia 4899 parasites per µl) were seen on blood smear examination along with infected red blood cells that were not enlarged and no gametocytes were seen. In the absence of Schüffner’s stippling, *P. vivax* and *P. ovale* infections were excluded. On re-examination of the smears at the Central Laboratory, which is a reference laboratory, the diagnosis of *Plasmodium* was confirmed. The laboratory staff was definite that the species was neither *P. vivax* nor *P. ovale*. The species resembled *P. malariae* with late band form trophozoites and basket forms, but the schizonts were not similar to *P. malariae* as they did not show the characteristic ‘daisy flower’ appearance. The few early trophozoite stages seen, including double chromatin stages, were similar to *P. falciparum.* Therefore, it was suspected that the species could be *P. knowlesi*. A genus and species-specific, nested polymerase chain reaction (PCR) assay [13] which was carried out at the Central Laboratory for the confirmation of diagnosis was positive for genus *Plasmodium,* while the species specific nested PCR was negative for *P. falciparum, P. vivax, P. ovale,* and *P. malariae.*

Initial blood tests revealed a white blood cell count of 4.2 K/µl (normal adult reference range 4.00–10.00), haemoglobin of 14.2 g/dl (normal reference range 11.0–16.0), platelets of 162 K/µl (normal ref range 150–450), a normal C reactive protein of 0.8 mg/l) and normal urea (21.8 mg/dl) and electrolytes.

In the absence of a definitive species diagnosis, considering the initial microscopy findings and based on WHO guidelines for the treatment of a suspected *P. knowlesi* case [14] whereby all suspicious or doubtful cases should be treated as *P. falciparum*, the patient was treated at the Teaching Hospital, Kandy as a *P. falciparum* infection, as per national guidelines, with the recommended dose of artemether (20 mg)-lumefantrine (120 mg) for a 3-day period and a single dose of primaquine (45 mg/kg body weight). No side effects were noted. No parasites were observed by microscopy and RDT by the third day (8 November) of treatment. The recovery period was uneventful. The patient was discharged with a diagnosis card with required dates for parasitological follow-up.

**Fig. 1:**
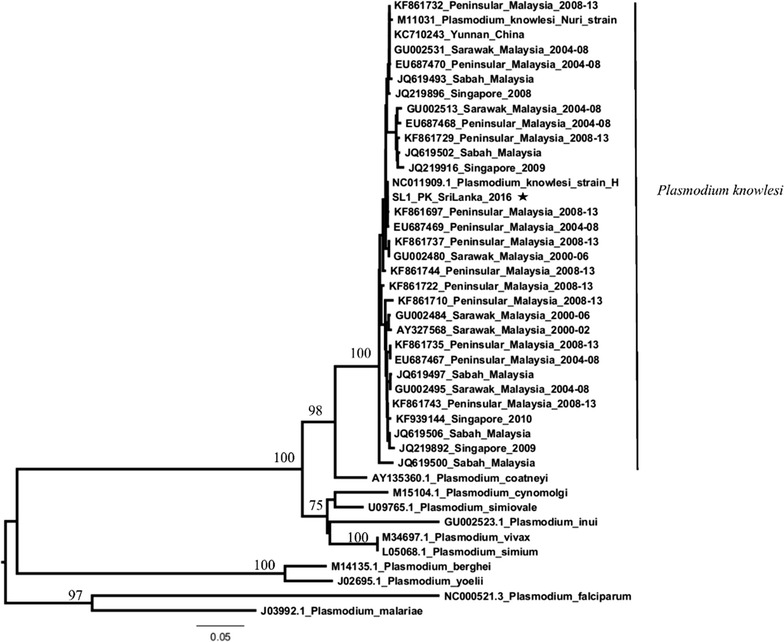
Phylogenetic analysis of *Plasmodium* species based on the non-repeat regions of *csp* gene. The tree was constructed using the neighbour-joining method implemented in MEGA version 6.0 software [17] under the Kimura-2 parameter model with transition and transversions. Figures on the main branches are bootstrap percentages obtained through 1000 replicates. The complete *csp* gene sequences retrieved from the GenBank database are shown in the tree with respective accession numbers, country of origin and the reported period (if available). *Plasmodium knowlesi* sequence of the case reported is marked with a *star*

Considering the disease epidemiology of malaria in Malaysia, to confirm the diagnosis of *P. knowlesi*, filter paper blood samples were sent to the Environmental Health Institute (EHI), National Environment Agency, Singapore, a public health laboratory for vector borne disease research and a WHO Collaborating Centre for Arboviruses and Related Vectors. The specimen was confirmed as positive for *P. knowlesi* by a semi-nested, species-specific PCR assay performed at EHI. The parasite was genetically characterized by sequencing the circumsporozoite (*csp*) gene. The phylogenetic analysis confirmed that the *P. knowlesi* strain reported in the current study was closely related to those reported previously from Peninsular Malaysia (Fig. 1). Therefore, analysis of parasite sequence data agreed with the case history that indicated a strong likelihood of acquiring the infection in Peninsular Malaysia.

### Confirmation of *P. knowlesi* by PCR technology

An in-house designed semi-nested PCR assay was used to confirm the presence of *P. knowlesi*. The first round (nest1) of PCR assay amplified the complete *csp* gene of *Plasmodium* spp. using oligonucleotides PkCSP-F [9] and PKCSPR2 [15]. PCR amplification was carried out in a 50 µl reaction, containing 5 µl of genomic DNA, 1× Phusion^®^ Flash PCR Master Mix (Thermo Fisher, USA) and 0.5 µM of each primer. The cycling parameters consisted of an initial denaturation at 98 °C for 10 s, followed by 44 cycles of 98 °C for 1 s, 55 °C for 5 s, and extension at 72 °C for 20 s. The final elongation was at 72 °C for 2 min. The first-round PCR assay products were subjected to a second round (nest 2) of PCR, using *P. knowlesi*-specific primers CspKnowlesiF (5′-ACCTTGARGTGGAAGCTTGTGT-3′) and PKCSPR2 [15]. The second-round amplification was carried out in a 20 µl reaction, containing 2 µl of the first-round PCR product as template, 1× reaction buffer, 2 mM MgCl_2_, 200 mM of each dNTP, 1.25 U of GoTaq DNA polymerase (Promega, USA), and 0.25 µM of each forward and reverse primer. The thermal cycling profile for the second-round PCR reaction was as follows; initial denaturation at 95 °C for 4 min, followed by 44 cycles of 95 °C for 30 s, annealing at 63 °C for 30 s, and extension at 72 °C for 30 s. The final elongation was at 72 °C for 2 min. All PCR reactions were carried out in a Veriti^®^ Thermal Cycler (Applied Biosystems, USA). The amplified products of the second-round PCR were visualized in 2% agarose gel stained with GelRed^®^ stain (Biotum Inc, USA).

### Cloning and sequencing of the *Plasmodium csp* gene

The complete *csp* gene of *Plasmodium* spp. amplified in the first-round PCR was cloned using the NEB^®^ PCR Cloning Kit (New England Biolabs, USA), according to the manufacturer’s instructions. Fourteen *Escherichia coli* transformants were screened by colony PCR. The colony PCR products were purified by using GeneJet^®^ PCR Purification Kit (Thermo Scientific, USA) according to the manufacturer’s protocol. The *csp* gene was sequenced at a commercial sequencing facility by using the Cloning Analysis Forward Primer (5′–ACCTGCCAACCAAAGCGAGAA-3′) and Cloning Analysis Reverse Primer (5′-TCAGGGTTATTGTCTCATGAGCG-3′) provided by the cloning kit. Sequencing was carried out according to the BigDye Terminator Cycle Sequencing kit (Applied Biosystems, USA) protocol.

### DNA sequence analysis

The consensus sequence of *csp* gene was obtained by assembling the raw sequencing data using Lasergene version 8.0 (DNASTAR, USA). The combined sequence encoding the non-repeat regions of N-terminal and C-terminal of the *csp* gene was aligned with the respective regions of *csp* gene sequences retrieved from the GenBank database using BioEdit version 7.0.9.0 [16]. Phylogenetic analysis was performed using the neighbour-joining method implemented in MEGA version 6.0 software [17]. The Kimura-2 parameter model with transition and transversion was used in the analysis. Internal node reliability was measured by the bootstrap method after 1000 replicates.

### Entomological and parasitological surveillance and vector control

A strenuous operation was mounted to carry out entomological and parasitological surveillance to ensure no transmission of the disease takes place. Entomological surveillance was commenced by the entomology team of the RMO of the district within 24 h of the case diagnosis. The patient was a resident of the Hanguranketha area in the Nuwara Eliya district where there is a potential for onward transmission as the primary vector for human malaria species in Sri Lanka, *Anopheles culicifacies* has been reported from the beds of rivers of Mahaweli and Uma Oya in this district [18]. As the only vectors known to transmit *P. knowlesi* belong to the Leucophyrus group [8, 19], receptivity of the areas surrounding the positive case was surveyed to determine the presence and distribution of potential vectors. Considering that *Anopheles elegans* and *Anopheles mirans* have been incriminated as a natural vectors of simian malaria in Sri Lanka [8, 20], and it has been shown to breed in the Central Province of the country [21], entomological surveillance activities were carried out to ensure no vector-borne transmission is taking place. Entomological techniques carried out included indoor hand collection, cattle-baited trap techniques, window traps, full and partial human landing night catches and larval surveys. During this survey, one *An. culicifacies* larva was encountered in a burrow pit (0.07 larvae per 100 dips), and *An. elegans* larvae (0.85 larvae per 100 dips) were found breeding in built wells in the areas situated within a 1-km radius surrounding the patient’s residence in the Nuwara Eliya district, but no adults were encountered. Entomological surveillance carried out in other sites at which the patient had stayed a night during the course of illness identified no anophelines.

Initiation of vector control activities took place immediately with the addition of larvivorous fish *Poecilia reticulata* (guppy) to wells, distribution of long lasting insecticide treated nets to 132 neighbouring households and fogging of the area with an insecticide mixture composed of d-tetramethrin 40 g/l, Cyphenothrin 120 g/l (Pesguard) EC to cover approximately 1-km radius of residence of the patient and all destinations he stayed additional nights.

When an imported malaria case is reported, all individuals who return with the index case from overseas are traced and each individual is tested for malaria. This patient had returned alone from Malaysia. In addition, 125 household members and neighbours of the case were screened for malaria by microscopy and RDT as part of the reactive case detection, and were negative for malaria. To ensure that there is no secondary transmission from the index case, parasitological screening of these close contacts was repeated 28 days after the diagnosis. In this survey, 152 blood smears were examined and all were negative for malaria.

### Malaria awareness programmes

A malaria awareness programme was carried out for the public in the area where the patient resides to educate the community on the need to seek treatment should they develop fever. The staff of all government and private hospitals and general practitioners in the vicinity were contacted to confirm the absence of any patients with malaria and to make them aware of the need to carry out a diagnostic test for malaria should a patient present with fever.

### Follow-up

As per guidelines of the AMC, the patient was screened for malaria by microscopy and RDT on the seventh and 14th days by a house visit at his residence. On the 21st day he reported to AMC headquarters and on the 28th and 42nd days he was screened at his work place in a separate district. The patient was negative for malaria during the follow-up period.

### Notification

The case was notified to the relevant administrative officers in the Central Province, and the higher officials of the Ministry of Health after confirmation of diagnosis in order to further strengthen malaria surveillance by the public and private sector health-care institutes.

The case was also notified to the Malaria Advisor, WHO SEARO and Coordinator Malaria and Vector borne and Parasitic Diseases Unit, WHO Regional Office of the Western Pacific. It was also discussed at the monthly review meetings of the AMC which are attended by all RMOs in the country, technical staff of AMC headquarters and representatives of the security forces. Representatives of the Sri Lanka Army, were advised to fill the gaps regarding prophylaxis to armed forces personnel going overseas to malaria endemic-countries.

## Discussion

Malaria transmission has been completely interrupted in Sri Lanka and locally acquired malaria cases are absent. A highly effective malaria surveillance system exists in the country, However, in spite of Ministry of Health’s efforts, both as a stand-alone organization as well as jointly with other medical professional bodies to train and re-train practising clinicians on the need to be alert to malaria, which remains a formidable challenge in the face of a ‘forgotten’ disease, this case was missed in the differential diagnosis by the two initial peripheral hospitals.

Malaria diagnosis is carried out free of charge in public health institutes, and private health care institutions can request malaria diagnosis directly either from AMC HQ or RMOs free of charge. Once diagnosed as malaria, even in the private sector as seen with this case, the case must be reported to the AMC. AMC provides anti-malarial drugs and guidance on malaria treatment. An immediate case investigation is initiated by obtaining information from each confirmed case to ascertain where and how the infection may have been acquired and the risks involved in the possibility of onward transmission. Travel history, regularity of chemoprophylaxis, patient’s behaviour to minimize mosquito contact while overseas and dates of all major events in the travel and clinical history are recorded both at the regional level and central level (AMC headquarters). The main focus of the AMC is to ensure that any malaria-positive case reported does not contribute towards development of an active focus which could be a challenge to the prevention of malaria re-introduction to Sri Lanka. The rapid response teams are mobilized by AMC headquarters and/or RMOs who are on constant vigil to carry out foci investigations around each positive case, targeted towards preventing secondary cases through onward transmission.

Microscopy has remained the gold standard for malaria diagnosis in Sri Lanka. Public health laboratory technicians working in over 200 hospital-based centres throughout the country have been trained by the AMC to carry out malaria microscopy free of charge, enabling early diagnosis and management of malaria cases. However, it is well-established that microscopy alone cannot reliably distinguish *P. knowlesi* from *P. malariae and P. falciparum* due to morphological similarities of the late stages (band forms) of *P. malariae* and *P. knowlesi* and the early stages (ring stages) of *P. falciparum* and *P. knowlesi* [22–24]. This is most likely the reason for the possible misdiagnosis of the case as a *P. falciparum* infection at the private hospital. It could be argued that due to the presence of trophozoites and schizonts which are not usually present in a *P. falciparum* infection, this could have been a mixed infection with *P. malariae*. Based on the morphology and history given by the patient, there was doubt at regional level and the Central Laboratory of AMC as to whether the species could be *P. knowlesi*. However, as *P. knowlesi* cases have never been reported in the country before, and since *P. knowlesi* and *P. malariae* cannot be differentiated by microscopy alone without molecular diagnostic confirmation, a species confirmation was not given by microscopic examination. The RDTs that are used to complement microscopic diagnosis of malaria in Sri Lanka can detect pan-specific pLDH, but to date no specific RDTs have been developed for the diagnosis of *P. knowlesi* infection and existing formats of RDTs are found to perform erroneously when used in *P. knowlesi*-endemic regions [25]. Therefore, PCR-based assays are recommended for the precise detection of *P. knowlesi* infections using species-specific primers [14]. The PCR assay conducted initially at the AMC excluded *P. falciparum, P. vivax, P. ovale,* and *P. malariae*. Given the strong suspicion of a diagnosis of *P. knowlesi*, assistance was sought from EHI, National Environmental Agency, Singapore, where the diagnosis of *P. knowlesi* was confirmed. The total duration from initial diagnosis of *Plasmodium* to the confirmation of *P. knowlesi* was 11 days. AMC Central Laboratory is now underway to establish the *P. knowlesi* specific PCR assay to advance its diagnostic capabilities.

This patient was managed as a case of *P. falciparum* infection, considering the need to treat for a worst case scenario as the laboratory evidence was inconclusive at the time of initiating treatment and also noting the fact that *P. falciparum* is prevalent in Malaysia [26]. The possible diagnosis of *P. knowlesi* was also evaluated considering (a) the high proportion of *P. knowlesi* infections (77.8% of malaria infections) in the Johor Bahru area in Malaysia from where this patient arrived [26]; (b) the strong suspicion of *P. knowlesi* based on the morphology of the blood smears; (c) the presence of a nested PCR positive for *Plasmodium* species. WHO guidelines [14] indicate that all suspicious or doubtful cases of *P. knowlesi* should be treated as *P. falciparum* and the artemether-lumefantrine combination used in this patient is highly effective for *P. knowlesi* [12]. Even though chloroquine is effective for the treatment of uncomplicated *P. knowlesi* infections, artemisinin based therapies are associated with a superior early therapeutic response [12]. In the absence of a definitive diagnosis following the completion of the full course of artemisinin based combination therapy, a single dose of primaquine was administered to this patient considering it as a possible *P. falciparum* infection. National treatment guidelines as well as WHO treatment guidelines [11] recommend using primaquine in *P. falciparum* infections as a measure of reducing transmission. However, primaquine is not considered necessary for the treatment of *P. knowlesi* infections in endemic regions as it is primarily a zoonosis without evidence of human to human transmission, the sexual stages are cleared with the drugs used to treat asexual stages and the fact that no hypnozoites are formed [27–29].

This patient was classified as an imported malaria case. This finding was further consolidated as: (a) there was no infective stages isolated during entomological surveillance; (b) no malaria positives being reported during the parasitological screenings; (c) no malaria positives reported from General Practitioners and clinicians serving in the areas where the case was reported from or had travelled to; (d) the patient would have been exposed to the bite of the vector which transmits *P. knowlesi* due to the nature of his occupation in the jungles of Malaysia; (e) chemoprophylaxis was not taken.

The natural macaque hosts of *P. knowlesi, Macaca fascicularis* and *Macaca nemestrina* are not found in Sri Lanka [30]. It is therefore unlikely that onward transmission of introduced or imported of *P. knowlesi* could occur or that the parasite could be maintained in the local monkey population. Thereby, any further onward transmission would have to be due to human to human transmission—which while experimentally proven to be possible [8], has not been demonstrated to occur naturally. In spite of extensive microscopical screening of close contacts, not finding any other person with a *P. knowlesi* infection also supports the fact that there is no human to human transmission.

The AMC rigorously implements malaria surveillance activities to keep Sri Lanka ‘malaria- free’. Regular screening programmes are conducted by the AMC amongst high-risk groups, including armed forces personnel returning from United Nations peace keeping missions to mitigate the risk of re-introduction of the disease. An excellent intersectoral collaboration exists with the armed forces to ensure that any individual arriving from a malaria-endemic country is informed in advance to the AMC. This patient was not screened for malaria as he arrived independently. AMC examines more than a million blood smears each year. With a proficient cadre of adequately resourced public health experts, parasitologists, entomologists, and malariologists and a good work ethic framework, the AMC currently strives to maintain its collaboration with diverse partners, including the military and professional medical associations to prevent a malaria resurgence in Sri Lanka.

## Conclusions

*Plasmodium knowlesi* should be suspected in patients returning from countries in the South Asian region where the parasite is prevalent and when blood smear results are inconclusive. The mechanisms in place with regard to rapid response mounted by the AMC when an imported malaria case is reported, can be replicated in other countries attempting malaria elimination and preventing re-introduction.

## Abbreviations

AMC: Anti Malaria Campaign
*csp*: circumsporozoite gene
EHI: Environmental Health Institute
pLDH: pan-Plasmodium antigen lactate dehydrogenase
PCR: polymerase chain reaction
RDT: rapid diagnostic test
RMO: Regional Malaria Officer
SEARO: Southeast Asia Regional Office
WHO: World Health Organization
